# The Impact of Patient Characteristics, Risk Factors, and Surgical Intervention on Survival in a Cohort of Patients Undergoing Neoadjuvant Treatment for Cervical Cancer

**DOI:** 10.3390/medicina59122147

**Published:** 2023-12-11

**Authors:** Irinel-Gabriel Dicu-Andreescu, Marian-Augustin Marincaș, Virgiliu-Mihail Prunoiu, Ioana Dicu-Andreescu, Sînziana-Octavia Ionescu, Anca-Angela Simionescu, Eugen Brătucu, Laurențiu Simion

**Affiliations:** 1 Clinical Department No 10, General Surgery, “Carol Davila” University of Medicine and Pharmacy, 050474 Bucharest, Romania; andreescugabriel43@gmail.com (I.-G.D.-A.);; 2 Department of Oncological Surgery, Oncological Institute “Prof. Dr. Alexandru Trestioreanu”, 022328 Bucharest, Romania; 3 Department of Obstetrics and Gynecology, Filantropia Clinical Hospital, 011132 Bucharest, Romania

**Keywords:** cervical cancer, lymph nodes, radiotherapy, radical hysterectomy, chemotherapy, stromal invasion, FIGO staging

## Abstract

*Introduction*: Cervical cancer is among the most frequent types of neoplasia worldwide and remains the fourth leading cause of cancer death in women, a fact that raises the necessity for further development of therapeutic strategies. NCCN guidelines recommend radiation therapy with or without chemotherapy as the gold standard for locally advanced cervical cancer. Also, some studies claim that performing surgery after chemo-radiation therapy does not necessarily improve the therapeutic outcome. This study aims to determine the impact of the risk factors, various characteristics, and surgical treatment for patients in different stages of the disease on survival rate. *Material and methods:* Our study started as a retrospective, observational, unicentric one, carried out on a cohort of 96 patients diagnosed with cervical cancer from the surgical department of the Bucharest Oncological Institute, followed from 1 January 2019 for a period of 3 years. After the registration of the initial parameters, however, the study became prospective, as the patients were closely monitored through periodical check-ups. The end-point of the study is either the death of the participants or reaching the end of the follow-up period, and, therefore, we divided the cohort into two subgroups: the ones who survived after three years and the ones who did not. All 96 patients, with disease stages ranging from IA2 to IIIB, underwent radio-chemotherapy followed by adjuvant surgery. *Results*: Among the 96 patients, 45 (46%) presented residual tumor after radio-chemotherapy. Five patients (5%) presented positive resection margins at the post-operative histopathological examination. The presence of residual tumor, the FIGO stage post-radiotherapy, positive resection margins, and lympho-vascular and stromal invasions differed significantly between the subgroups, being more represented in the subgroup that reached the end-point. Variables correlated with the worst survival in Kaplan–Meier were the pelvic lymph node involvement—50% at three years (*p*—0.015)—and the positive resection margins—only 20% at three years (*p* < 0.001). The univariate Cox model identified as mortality-associated risk factors the same parameters as above, but also the intraoperative stage III FIGO (*p* < 0.001; HR 9.412; CI: 2.713 to 32.648) and the presence of post-radiotherapy adenopathy (*p*—0.031; HR: 3.915; CI: 1.136 to 13.487) identified through imagistic methods. The independent predictors of the overall survival rate identified were the positive resection margins (*p*—0.002; HR: 6.646; CI 2.0 to 22.084) and the post-radiotherapy stage III FIGO (*p*—0.003; HR: 13.886; CI: 2.456 to 78.506). *Conclusions*: The most important predictor factors of survival rate are the positive resection margins and the FIGO stage after radiotherapy. According to the NCCN guidelines in stages considered advanced (beyond stages IB3, IIA2), the standard treatment is neoadjuvant chemoradiotherapy. In our study, with radical surgery after neoadjuvant therapy, 46% of patients presented residual tumor at the intraoperative histopathological examination, a fact that makes the surgical intervention an important step in completing the treatment of these patients. In addition, based on the patient’s features/comorbidities and the clinical response to chemotherapy/radiotherapy, surgeons could carefully tailor the extent of radical surgery, thus resulting in a personalized surgical approach for each patient. However, a potential limitation can be represented by the relatively small number of patients (96) and the unicentric nature of our study.

## 1. Introduction

Worldwide, approximately 570,000 women are diagnosed with cervical cancer each year, which is the fourth leading cause of cancer death in women after breast, lung, and colorectal cancer [1]. However, in developing countries, it rises to the first position due to insufficient screening caused by lower socio-economic status or limited access to healthcare [2]. It is caused by certain strains of the human papillomavirus (HPV). There are more than 120 HPV genotypes identified, of which 15 can cause cervical cancer. The majority of HPV infections, about 90%, are neutralized by the body’s immune system within one to two years after the infection [3,4].

However, there are a lot of additional risk factors for cervical cancer that have a great potential to influence the risk of contracting HPV infection, such as by increasing the exposure, which includes early initiation of sexual activity and having multiple sexual partners [5], or the ability to fight against the infection by weakening the immune system, which can be represented by tobacco use, co-infection with HIV/AIDS, the use of immunosuppressive medications, or a low fruit and vegetables diet [6,7,8]. 

Some studies have also suggested that long-term use (five or more years) of oral contraceptives may slightly increase the risk of cervical cancer. However, this risk decreases after stopping the use [9]. In addition, some research has shown a potential link between long-term Chlamydia infections and an increased risk of cervical neoplasia, possibly due to the chronic inflammation induced [10]. In Romania, there are 4343 new cases of cervical and endometrial cancer every year and 1909 deaths caused by this disease; these figures represent 7% of the total number of cases in Europe. Also, Romania ranks first in the European Union in terms of cervical cancer mortality—the mortality rate is four times higher than the European Union average.

According to recent studies, although an increased percentage of Romanian women understand the importance of screening and the benefits of early diagnosis, a large proportion of them postpone routine check-ups due to a lack of time and financial resources. Moreover, the only free screening program implemented in Romania between 2012 and 2017 was a failure due to poor quality procedures, low number of women tested, underfunding, and lack of information and promotion [11].

For early stages of cervical cancer (stages IA1-IA2), the NCCN guidelines recommend cervical conization, or trachelectomy or radical hysterectomy with or without pelvic lymph node biopsy depending on the patient’s option to preserve fertility or not. From stages IB1 to IIA1, before the surgical intervention, external radiotherapy with or without chemotherapy is recommended. After stage IIA2, the tumor is considered loco-regionally advanced and only radiation therapy with sensitizing chemotherapy is recommended, and, in selected cases, completion with radical surgery.

Although Romania is trying to implement and comply with international diagnostic and treatment guidelines, due to the advanced stage of cancer at the time of the presentation of the patients and the often-limited availability of resources, a personalized therapeutic approach is needed. This can be achieved by a thoughtful analysis of the patients and their response to chemo-radiotherapy as neo-adjuvant therapy. Moreover, the integration of multidisciplinary teams could help prevent or attenuate post-operative morbidities, therefore preserving women’s quality of life.

The guide of the Bucharest Oncological Institute (IOB) “Prof. Dr. Alexandru Trestioreanu” follows the European and the international NCCN treatment guidelines [12], but it also provides an alternative for selected cases in which it is considered that the optimal radiation dose, varying between 54 and 63 Gy, combined with chemotherapy will not succeed in eliminating the tumor cells from the cervix. In these cases, the first steps of the treatment are concurrent radio-chemotherapy, similar to that proposed by the guidelines—external irradiation, intracavitary brachytherapy, and cisplatin-based chemotherapy. This step is followed after 6 to 8 weeks by surgical intervention, which consists of Type III radical hysterectomy with pelvic lymphadenectomy with or without para-aortic lymph node sampling or para-aortic lymphadenectomy [13].

The aim of our study was to determine the impact of the risk factors and the various characteristics of patients in different stages of the disease on survival rate and also to determine the role of surgical intervention even in advanced stages. We focused on this last aspect because it can offer another perspective to the classical treatment limited only to a combination of chemo- and radiotherapy.

## 2. Materials and Methods

Our study started as a retrospective, observational, unicentric one, carried out on a cohort of 96 patients diagnosed with cervical cancer in the surgery department of the Bucharest Oncological Institute, “Prof. Dr. Alexandru Trestioreanu”(IOB) followed from 1 January 2019—the year in which the patients underwent surgery, for a period of three years, until 1 January 2022. We included in the study all the patients who were diagnosed with cervical neoplasia and underwent neoadjuvant treatment, starting with the International Federation of Gynecology and Obstetrics 2018 (FIGO) stage IA up to stage IIIB. Each case was evaluated after the completion of neoadjuvant radio-chemotherapy, and after six weeks, the surgical intervention was performed (Type III radical hysterectomy with pelvic lymphadenectomy).

Initially, we selected 150 patients, the total number of patients with cervical cancer operated on in 2019. However, 25 patients were excluded from the study due to their option to be followed up in regional medical institutions due to long travel distances and financial reasons, and we also excluded 29 of them due to the lack of presentations at periodical check-ups (most likely for similar financial reasons). In addition, we excluded those under 18 years old and those with neoplastic pathology of a different location (synchronous neoplasia).

We have included patients with cervical neoplasm from stages IA2 to IIIB who underwent Type III radical hysterectomy with pelvic lymphadenectomy in the year 2019 in our department based on our local guidelines. We selected patients from both urban and rural areas with cervical cancer, often presented in advanced stages considered beyond the operable stages (beyond stages IB3, IIA2) with centrally localized disease, and who subsequently underwent radio-chemotherapy treatment. After the neoadjuvant treatment, the patients were reevaluated by imagistic methods to evaluate the dimensional regression of the tumor and the status of the iliac and lumbo-aortic lymph nodes. After 6 weeks from the end of the neoadjuvant treatment, the radical surgical intervention was performed.

In most cases, surgery consisted of Type III radical hysterectomy with pelvic lymphadenectomy. All patients were followed for at least three years, the end-point being represented by the death of the patient if this occurred before the end of the follow-up or by the end of this time frame. After the registration of the initial parameters, however, the study became prospective, as the patients were closely monitored through periodical check-ups. Based on medical records, complete data about the patient’s characteristics, clinical examination, laboratory tests, personal history of abdominal surgery, MRI evaluation, the histological type of the cervical tumor identified by biopsy, FIGO stages pre- and post-radiotherapy, as well as intraoperative FIGO stage, the number of sessions, and the dose of radiotherapy (between 54 and 63 Gy) were registered, as well as the need for sensitizing chemotherapy sessions; it was also recorded whether or not the patients required adjuvant chemotherapy.

The presence of pre- and post-radiotherapy adenopathy, and also the presence of parametrial invasion, were initially evaluated through imagistical methods. The stromal invasion, the residual tumor size (after the neoadjuvant treatment), the lymphovascular and the perineural invasion, the presence of parametrial invasion, the positivity of resection margins, and the lymph nodes sampled were also evaluated intra- and post-operatively through anatomopathological examination. Last but not least, we recorded post-operative complications and confirmed the survival status at three years by periodic check-ups.

The outcomes were the impact of patient characteristics and variables that increase the risk of death in relation to independent predictors of survival. We compared our approach with the survival rate reported in the literature to determine the differences between the classic approach recommended by the guidelines, which limits the therapy to radio- and chemotherapy, and the one practiced in the Surgery Clinic of IOB, which completes radio-chemotherapy with surgical intervention.

For the statistical analysis, we used the SPSS program. To evaluate the distribution of continuous variables between the two groups, given that they do not have a normal distribution, the Mann–Whitney U test was used. To test the differences between categorical and nominal variables, we used Fisher’s exact test.

## 3. Results

Below, we detailed the characteristics of the patients included in the study divided into two groups—those alive at three years and those who died before reaching this endpoint. Age and hemoglobin were normally distributed and are, therefore, reported as mean and standard deviation (SD). All the other variables had an abnormal distribution and are reported as frequency, median, and inter-quartile range (IQR) (Table 1, Table 2, Table 3 and Table 4).

### 3.1. Differences between the Patients Alive after 3 Years and Those Who Died

As a first step, we tried to find if there were statistically significant differences between patients who died and those who remained alive at 3 years. These tests showed that the FIGO stage post-radiotherapy, the residual tumor size, positive resection margins, and lympho-vascular and stromal invasions differed significantly between the subgroups, being more represented in the subgroup that did not survive at three years of follow-up (Table 5).

### 3.2. Differences between FIGO Stages Evaluated Pre- and Post-Radiotherapy and Intraoperatively

As a second step, we were interested in finding how the FIGO stage changes pre-radiotherapy versus post-radiotherapy versus the stage found intraoperatively. To see the differences in evolution, we used the Wilcoxon Signed Rank Test. The result, as was expected, was that the post-radiotherapy FIGO stage regressed significantly—*p* < 0.001, which confirms the importance of neoadjuvant radiotherapy. Also, the intraoperative FIGO stage was reported to be lower than that found post-RT, with *p* = 0.013.

### 3.3. Rate of Survival

To assess the rate of survival, Kaplan–Meier survival curves were constructed in the first step and, subsequently, Cox models. Also, to increase the accuracy of the results, the FIGO stage has been simplified as follows: stages IA and IB have become stage 1, stages IIA and IIB have become stage 2, and stages IIIA and IIIB have become stage 3; the absence of tumor was coded as stage 0.

#### 3.3.1. Survival Rate in Correlation with Post-RT FIGO Stage

**Figure 1:**
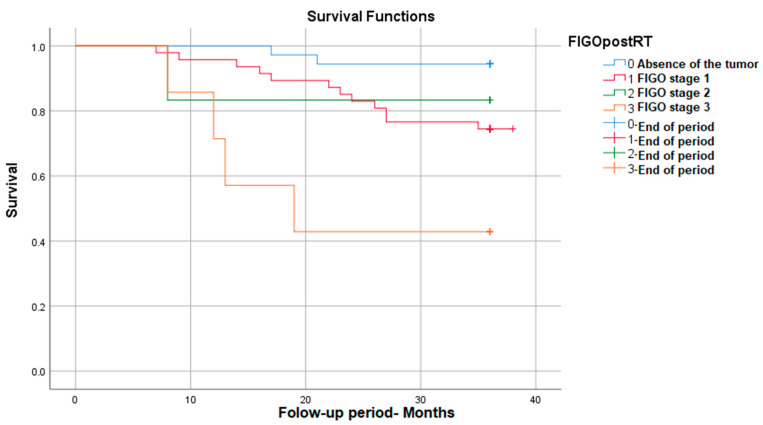
Survival rate in correlation with post-RT FIGO stage.

The survival was significantly different depending on the post-radiotherapy FIGO stage, with it decreasing with the increase in the FIGO stage: *p* (Log Rank) = 0.002. As can be seen in the figure below, for FIGO stage I, post-RT survival reaches 95%, and for advanced stages (stage III FIGO), survival decreases to 42% (Figure 1).

#### 3.3.2. Survival Rate in Correlation with Intraoperative FIGO Stage

**Figure 2:**
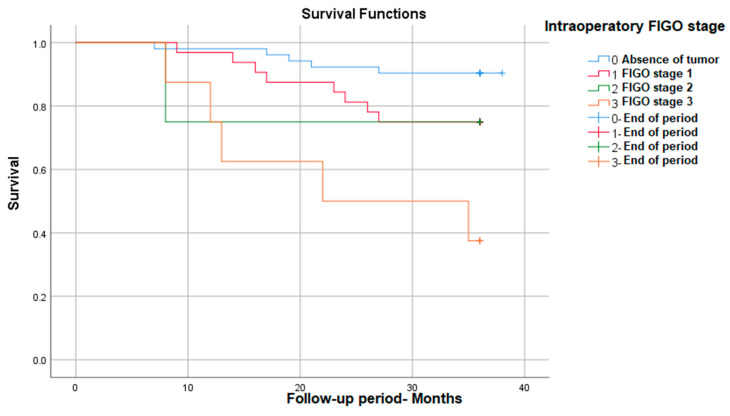
Survival rate in correlation with intraoperative FIGO stage.

We also found that between the FIGO stages evaluated during the surgical intervention, there are statistically significant survival differences, with stage 3 having the lowest survival at 3 years (38%)—*p* (Log Rank) = 0.001 (Figure 2).

#### 3.3.3. Survival Rate in Correlation with Stromal Invasion

**Figure 3:**
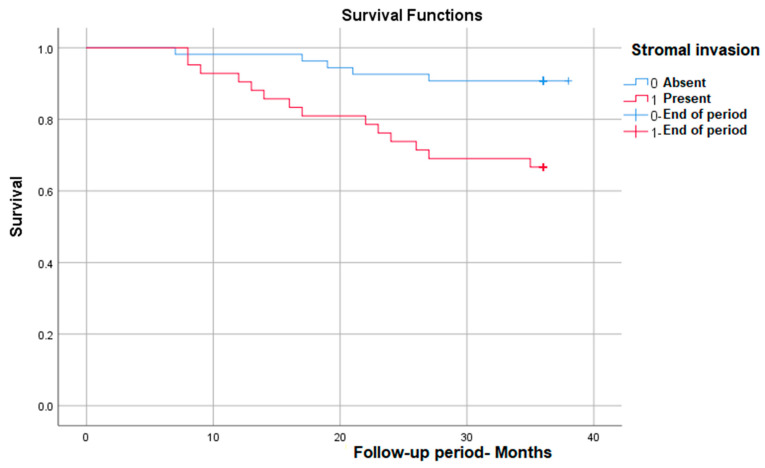
Survival rate in correlation with stromal invasion.

As we expected, stromal invasion is correlated with a significantly lower survival rate in those in which it is present, from nearly 93% in patients without invasion to 65% in those with invasion—*p* (Log Rank) = 0.003 (Figure 3).

#### 3.3.4. Survival Rate in Correlation with Lympho-Vascular Invasion

**Figure 4:**
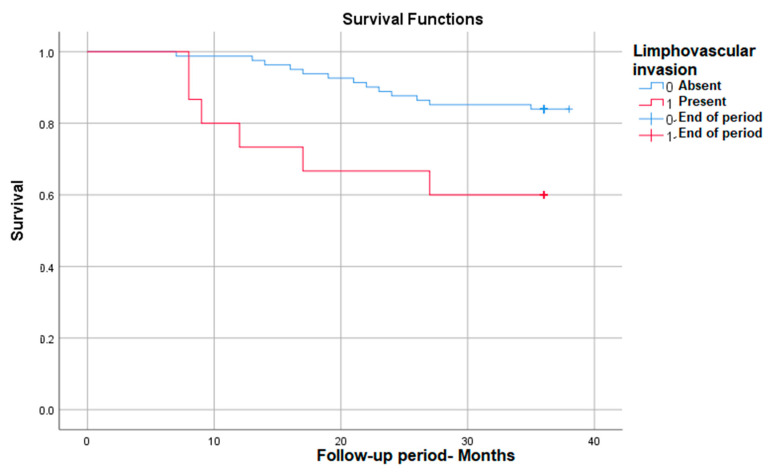
Survival rate in correlation with lympho-vascular invasion.

Also, the lympho-vascular invasion correlates with significantly lower survival in those in which it is present, down to 60%—*p* (Log Rank) = 0.015 (Figure 4).

#### 3.3.5. Survival Rate in Correlation with Positive Resection Margins

**Figure 5:**
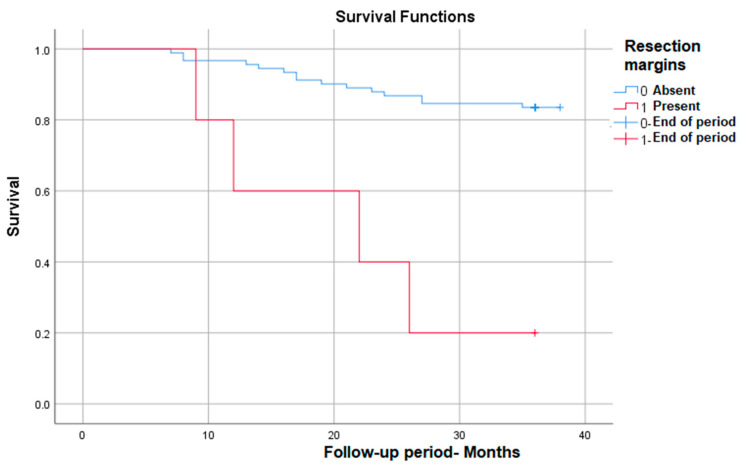
Survival rate in correlation with positive resection margins.

Positive resection margins correlate with the most markedly decreased survival (from 83% to only 20% at 3 years) in those in which they are present—*p* (Log Rank) < 0.001 (Figure 5).

#### 3.3.6. Survival Rate in Correlation with Lymph Node Invasion

**Figure 6:**
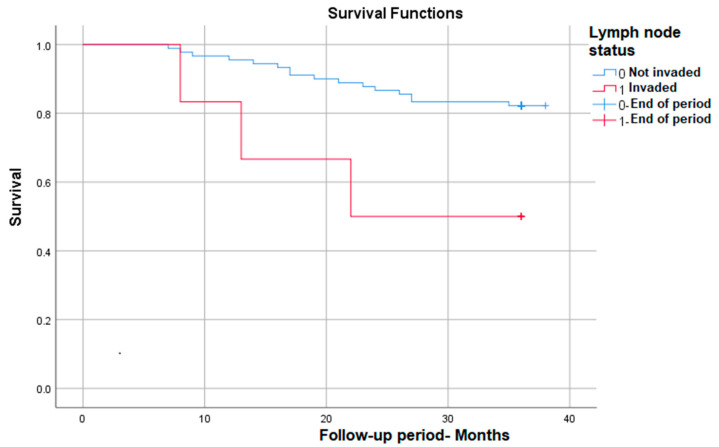
Survival rate in correlation with lymph node status.

Lymph node invasion identified post-operatively (implicitly post-radiotherapy) also correlates with significantly lower survival at nearly 50% compared with patients without lymph node invasion—*p* (Log Rank) = 0.022 (Figure 6).

#### 3.3.7. Survival Rate in Correlation with the Presence of Residual Tumor after Neoadjuvant Treatment

**Figure 7:**
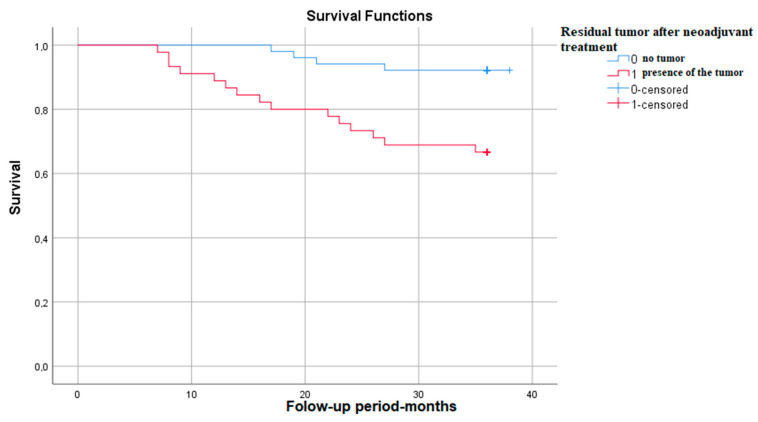
Survival rate in correlation with the presence of residual tumor.

The Kaplan–Meier survival graph in patients with residual tumor after neoadjuvant treatment shows that survival at 3 years is approximately 65%—*p* (Log Rank)—0.001 (Figure 7).

It should be noted that the histological type of the tumor, pre-radiotherapy FIGO stage, perineural invasion, and extension in the parameters were monitored, but the statistical results were not significant.

#### 3.3.8. Frequency of the Residual Tumor in Correlation to the FIGO Stages

**Figure 8:**
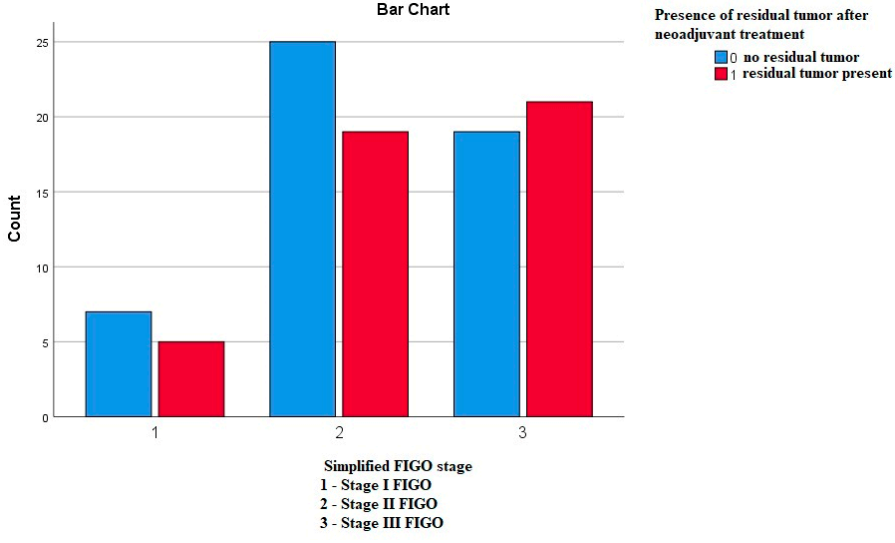
Frequency of the residual tumor in correlation to the FIGO stages.

Using the Pearson’s chi-square test, we compared whether the frequency of the residual tumor differs according to the FIGO stages, and we did not obtain any statistically significant difference—*p*—0.644 (Figure 8).

### 3.4. Variables That Are Significantly Associated with An Increased Risk of Death

To assess this, we tested each variable in a univariate Cox model, which returned the following results: the presence of post-radiotherapy adenopathy, and also the post-radiotherapy and intraoperative FIGO stage, the stromal invasion, the presence of residual tumor (after neoadjuvant treatment), the lympho-vascular invasion, the positive resection margins, the size of initial tumor, and, last but not least, the intraoperative nodal status were associated with an increased risk of reaching the end-point. The exact statistical values are presented in Table 6.

### 3.5. Independent Predictors of Survival

We introduced all the variables significantly associated with the risk of death in a multivariate Cox model, which showed that the independent predictors of survival were found to be the positive resection margins and the post-radiotherapy stage 3 FIGO. This aspect is of particular importance in daily practice because, in contrast to the post-radiotherapy FIGO stage, which cannot be completely controlled, the response is at least partially different from one patient to the other, and the positivity of the resection margins can be prevented by a wider intraoperative approach when it is possible (Table 7).

In contrast, in the multivariate Cox model, the presence of residual tumor after radio-chemotherapy was not an independent predictor of survival.

## 4. Discussions

The motivation of our study was to explore the characteristics and the risk factors for a lower survival rate in patients with cervical cancer who underwent neoadjuvant treatment completed by surgical intervention [14]. We opted for this approach because our local guidelines and experience argued a better control of the advanced disease. We consider it as a very strong point and also a particularity that our study allowed us to explore the real histopathological response to the classical therapeutic approach—which is limited to chemo-radiotherapy. The results obtained were surprising—out of 96 patients, 45 of them (46%) presented residual tumor after neoadjuvant treatment, demonstrated by post-operative histopathological examination.

Taking into consideration that the patients received the neoadjuvant treatment in doses recommended by international guidelines, it means that it failed to completely destroy tumor cells in 46% of cases. Moreover, this finding was also confirmed by another study carried out in our institute on a different cohort, which evaluated the response to neoadjuvant treatment according to the tumor histological type [15]. In that cohort, 55% of the patients had residual tumor [15]. Because of these results, we can claim that the surgical intervention is still of value, even in advanced stages of disease, by providing better local control.

Another fact that should be mentioned is that in our cohort, out of a total of 96 patients, 85 (86%) were in advanced stages, with no statistically significant differences between the frequency of residual tumor and the exact FIGO stage. However, the patients who had residual tumors on the post-operative resection piece had a five-times higher risk of death (and overall survival at three years of only 68%), which is to be expected since this means that they had a poorer response to the neoadjuvant treatment. This survival rate is also supported by other studies that obtained similar results in patients with residual tumors who underwent hysterectomy as adjuvant treatment after chemo-radiotherapy [16,17]. On the other hand, because of the low number of patients, it can be argued that further studies are needed in order to definitively confirm these results.

We also aimed to identify the survival predictors in cervical cancer, as they can provide valuable information for healthcare professionals and patients about the potential outcome of the disease and guide treatment decisions [14]. The first is the FIGO stage, in which the disease is diagnosed. According to the literature, for patients in early stages, like IB (IB1, IB2, and IB3) on which neoadjuvant and adjuvant treatment were performed, survival is up to 95% and decreases gradually down to 42% in stage III FIGO [18,19]. In our study, the survival rate at three years was 88.5% in stage I (IA and IB), 88.4% in stage II (IIA and IIB), and 68.4% in stage III (IIIA and IIIB). This means that we obtained better survival for advanced stages of cancer on which radio-chemotherapy and surgery were performed.

The size of the initial tumor represents another factor with a major impact on survival. In general, between mortality and the size of the tumor is a directly proportional relationship, but if a favorable response to neoadjuvant therapy is obtained, the survival rate increases [20,21]. In our study, however, this parameter did not appear to be statistically significant, but these results may be influenced by the small number of patients.

Lymph node invasion is the main sign that the cancer has spread and represents an independent criterion for establishing the stage of the disease [22]. In our study, lymph node invasion also represents one of the significant factors associated with an increased risk of death, without being an independent factor, however. According to data from the literature, the presence of adenopathy identified by imaging or confirmed by biopsy is associated with a significantly lower survival rate [23].

Also, the histological type of cancer is an important predictor of survival, as different histological forms can have a specific evolution rate and a different response to neoadjuvant and adjuvant treatment. Some recent studies showed that patients with adenocarcinoma or adenosquamous carcinoma had a significantly weaker response to treatments such as chemoradiotherapy than those with squamous cell carcinoma [15,24]. In our study, on the other hand, 88% of the patients had squamous cell carcinoma, and the statistical results showed that survival does not vary significantly in relation to the other histological types.

The age at which the disease is diagnosed can also influence survival, with advanced age being associated with other comorbidities that can influence treatment options and the general condition of patients. According to the American Cancer Society (ACS), women who are 65 or older have a 46% relative survival rate, while women between the ages of 50 and 64 have a 61% relative survival rate [25]. The best survival rate is for women between 35 and 50 years, who have a 77% relative survival rate [25]. Surprisingly, women who are under 35 years and have advanced loco-regional disease have a worse prognosis compared to the elderly, a fact that can be explained by the psycho-social impact, which is the greatest in this category of age [25,26]. In our study, the average age of the patients was 57 years and does not represent a statistically significant variable.

The status of the resection margins is also very important. According to the literature, negative margins are associated with increased survival [27,28]. This aspect was also confirmed in our study, and we also found that it represents an independent predictor factor. Being a preventable risk factor, the positivity of the resection margins can be avoided by using a careful surgical technique and an experienced team.

Socio-economic factors, such as access to medical services, social status, and environment, can influence survival, as patients from poor backgrounds tend, as expected, to present in more advanced stages of the disease, and their adherence to neoadjuvant and adjuvant treatment is lower [29]. In our study, 67% of patients came from rural areas, but there were no statistically significant differences compared to those from urban areas. An explanation can be the fact that presentation for regular check-ups in Romania is precarious regardless of the environment [30]. People who live in remote places usually have difficulties accessing medical or screening services, have poor living conditions, and have a lower level of education. Also, especially in rural areas, women tend to consider genital pathology as a shameful subject and often wait until discomfort or bleeding occurs.

Regarding the surgical intervention, it was performed through an open route, the minimally invasive option not being available in our center due to the lack of technical facilities. However, according to some studies, there are no significant differences regarding recurrence between the minimally invasive and open techniques under the condition that the patients had received neoadjuvant pre-operative treatment [31,32]. In addition, radical surgical treatment applied 6 weeks after neoadjuvant treatment can be an independent factor in reducing mortality due to relapse of cancer [33].

## 5. Conclusions

Surgery plays an important role in the management of cervical cancer. Type III radical hysterectomy with bilateral pelvic lymph node dissection using the open route is the standard surgical procedure for early cervical cancer. For advanced disease, the recommended standard treatment is currently represented by concomitant chemoradiotherapy. Despite the recent improvements related to these therapeutic strategies, the long-term overall survival rate still ranges from about 57% to 67%. Local recurrence and distant metastasis remain the main causes of therapeutic failure in cervical cancer, proving that the microscopic residual disease, often undetectable with current diagnostic strategies, represents the major risk factor for treatment failure.

The analysis of our cohort followed between 2019–2022 showed that there are significant differences between the group of those who survived after three years of follow-up and those who did not. Among the main differences are the FIGO stage and the presence of the residual tumor after neoadjuvant treatment—more advanced in those who died—and the lympho-vascular and stromal invasions, which, again, were significantly more frequent in those who did not survive until the end of the follow-up. Out of 96 patients, 45 of them (46%) presented residual tumors on the excision specimen after neoadjuvant treatment and only 3% of these presented lymph node metastases on anatomopathological examination.

Using the univariate Cox model, we found that the presence of post-radiotherapy lymph nodes, post-radiotherapy FIGO stages I and III, intraoperative FIGO stage III, the presence of stromal invasion, lympho-vascular invasion, presence of residual tumor, positive resection margins, and nodal involvement were associated with increased risk of death. Most importantly, the two independent predictors of three-year survival are the presence of positive resection margins and post-radiotherapy FIGO stage III, and even though we cannot control the stage in which our patient presents nor their response to the treatment, we can make efforts to prevent the positivity of the resection margins and, therefore, increase the chances of survival.

We found that at three years, depending on the FIGO stage at the presentation, the survival rate was 88.5% in stage I (IA and IB), 88.4% in stage II (IIA and IIB), and 68.4% in stage III (IIIA and IIIB). These findings are of much interest because not only did we obtain a very good survival in advanced stages (up to 20% compared to the data from the literature), but we also showed that the combination of neoadjuvant treatment and surgical intervention could be a valuable option that should be taken into consideration in many more cases, which is a strength of our study.

We consider it a strength that our study allowed us to evaluate survival according to the described factors, such as the imaging staging before and after neoadjuvant radio-chemotherapy. In addition, we completed the findings with the information gathered intraoperatively, a fact not that common since, in other centers, the surgical intervention is no longer practiced after neoadjuvant treatment. Future perspectives in the treatment of cervical cancer can be represented by the transition from the one-size-fits-all principle to a personalized treatment. A weakness of the study is the relatively low number of patients and the three-year period in which the patients were evaluated, taking into account that most studies on survival in cervical cancer are carried out over a period of five years. However, because the patients were closely monitored and the stage of the disease was also advanced in the majority of the cases, it was considered an optimal follow-up period, and our results can serve as a foundation for further research.

## Figures and Tables

**Table 1:** General characteristics of the patients.

	Total (96)	Deceased at 3 Years (19)	Alive at 3 Years (77)
**Age, years, median (SD)**	57.14 (11.7)	57.16 (13.6)	57.13 (11.3)
**Environment, n (%)**	urban: 31 (32) rural: 65 (67)	urban: 6 (31) rural: 13 (68)	urban: 25 (32) rural: 52 (67)
**Personal history of abdominal surgery, n (%)**	12 (12)	2 (10)	10 (12)
**Pre-operative hemoglobin, median (DS)**	12.05 (1.5)	11.66 (1.5)	12.15 (1.5)
**Pre-operative leukocyte count, median (IQR)**	5780 (2300)	5500 (2090)	5800 (2410)

SD—standard deviation; n—number; IQR—inter-quartile range; RT—radiotherapy.

**Table 2:** Pre-RT and post-RT staging and pre-operative histology.

	Total (96)	Deceased at 3 Years (19)	Alive at 3 Years (77)
**Pre-RT FIGO stage, n (%)**	IA: 3 (3)	IA: 0 (0)	IA: 3 (3)
	IB: 9 (9)	IB: 2 (10)	IB: 7 (9)
	IIA: 16 (16)	IIA: 2 (10)	IIA: 14 (18)
	IIB: 28 (29)	IIB: 3 (15)	IIB: 25 (32)
	IIIA: 3 (3)	IIIA: 1 (5)	IIIA: 2 (2)
	IIIB: 37 (38)	IIIB: 11 (57)	IIIB: 26 (33)
**Histological form of cancer (pre-operative biopsy), n (%)**	1. Squamous cell carcinoma—85 (88)	1. Squamous cell carcinoma—16 (84)	1. Squamous cell carcinoma—69 (89)
	2. Adenocarcinoma—8 (88)	2. Adenocarcinoma—2 (10)	2. Adenocarcinoma—6 (7)
	3. Adenosquamous carcinoma—3 (3)	3. Adenosquamous carcinoma—1 (5)	3. Adenosquamous carcinoma—2 (2)
**Initial tumor size, median (IQR)**	0 (1)	1 (2)	0 (1)
**Presence of demarcation limits at imaging pre-radiotherapy, n (%)**	66 (68)	11 (57)	55 (71)
**Parametrial invasion pre-radiotherapy, n (%)**	43 (44)	11 (57)	32 (41)
**Post-RT FIGO, n (%)**	IA: 11 (11)	IA: 2 (10)	IA: 9 (11)
	IB: 36 (37)	IB: 10 (52)	IB: 26 (33)
	IIA: 4 (4)	IIA: 1 (5)	IIA: 3 (3)
	IIB: 2 (2)	IIB: 0 (0)	IIB: 2 (2)
	IIIA: 1 (1)	IIIA: 1 (5)	IIIA: 0 (0)
	IIIB: 6 (6)	IIIB: 3 (15)	IIIB: 3 (3)
	Absence of tumor: 36 (37)	Absence of tumor: 2 (10)	Absence of tumor: 34 (44)
**Post-RT Lymphadenopathy, n (%)**	6 (6)	3 (15)	3 (3)

n—number; IQR—inter-quartile range; FIGO—International Federation of Obstetrics and Gynecology; RT—radiotherapy.

**Table 3:** Neoadjuvant and adjuvant radio-chemotherapy.

	Total (96)	Deceased at 3 Years (19)	Alive at 3 Years (77)
**RT dose, median (IQR)**	50 (0.3)	50 (0.4)	50 (0)
**Nr. of RT sessions, median (IQR)**	25 (0)	25 (2)	25 (0)
**Sensitization chemotherapy, median (IQR)**	5 (5)	5 (5)	5 (5)
**Post-operative chemotherapy, n (%)**	21 (21)	7 (36)	14 (18)

IQR—inter-quartile range; RT—radiotherapy.

**Table 4:** Intraoperative staging and histology.

	Total (96)	Deceased at 3 Years (19)	Alive at 3 Years (77)
**Intraoperative FIGO, n (%)**	IA: 11 (11)	IA: 2 (10)	IA: 9 (11)
	IB: 21 (21)	IB: 6 (31)	IB: 15 (19)
	IIA: 2 (2)	IIA: 1 (5)	IIA: 1 (1)
	IIB: 2 (2)	IIB: 0 (0)	IIB: 2 (2)
	IIIA: 1 (1)	IIIA: 1 (5)	IIIA: 0 (0)
	IIIB: 7 (7)	IIIB: 4 (21)	IIIB: 3 (3)
	Absence of tumor: 51 (53)	Absence of tumor: 4 (21)	Absence of tumor: 47 (61)
**Intraoperative histological form, n(%)**	1. Squamous cell carcinoma—32 (33)	1. Squamous cell carcinoma—10 (52)	1. Squamous cell carcinoma—22 (28)
	2. Adenocarcinoma—8 (8)	2. Adenocarcinoma—3 (15)	2. Adenocarcinoma—5 (6)
	3. Adenosquamous carcinoma—2 (2)	3. Adenosquamous carcinoma—0 (0)	3. Adenosquamous carcinoma—2 (2)
	4. In situ carcinoma—3 (3)	4. In situ carcinoma—2 (10)	4. In situ carcinoma—1 (1)
**Stromal invasion, n (%)**	42 (43)	14 (73)	28 (36)
**Lymphovascular invasion, n (%)**	15 (15)	6 (31)	9 (11)
**Perineural invasion, n (%)**	5 (5)	2 (10)	3 (3)
**Intraoperative parametrial extension, n (%)**	5 (5)	2 (10)	3 (3)
**Positive resection margins, n (%)**	5 (5)	4 (21)	1 (1)
**Residual tumor**	45 (46)	15 (79)	30 (39)
**Positive intraoperative lymph nodes, n (%)**	6 (6)	3 (15)	3 (3)
**Number of nodes sampled, median (IQR)**	5 (4)	5 (7)	5 (4)
**Number of metastatic nodes, median (IQR)**	0 (0)	0 (0)	0 (0)
**Number of post-operative complications, n (%)**	19 (19)	3 (15)	16 (20)

n—number; IQR—inter-quartile range; FIGO—International Federation of Obstetrics and Gynecology.

**Table 5:** Differences between the two subgroups.

Variable	*p* Value
**Age**	0.87
**Environment**	0.96
**Hemoglobin**	0.26
**No. leukocytes**	0.25
**Histological type of cancer**	0.45
**RT dose**	0.31
**No. RT sessions**	0.13
**Sensitization chemotherapy**	0.91
**Post-RT adenopathy**	0.055
**Histological form of cancer**	0.76
**FIGO pre-RT**	0.36
**FIGO post-RT**	0.028
**IRM demarcation plan**	0.25
**Parametric invasion MRI**	0.20
**Initial tumor size**	0.002
**Stromal invasion**	0.004
**Lymphovascular invasion**	0.043
**Perineural invasion**	0.24
**Extension in parameters**	0.24
**Positive resection margins**	0.001
**Nodal status**	0.055
**No. nodes sampled intraoperatively**	0.86
**No. metastatic nodes**	0.06
**Residual tumors**	0.002

No—number; RT—radiotherapy; FIGO—International Federation of Obstetrics and Gynecology.

**Table 6:** Variables associated with increased risk of death.

Variable	*p* Value	HR	CI
The presence of post-RT adenopathy *	0.031	3.915	1.136—13.487
FIGO stage post-RT **	Stage I—0.035 Stage III—0.001	Stage I—4.987 Stage III—16.893	Stage I—1.116–22.288 Stage III—3.076–92.759
**Intraoperative FIGO stage III**	<0.001	9.412	2.713–32.648
**Stromal invasion**	0.007	4.123	1.484–11.455
**Initial tumor size**	<0.001	1.401	1.167–1.682
Lymphovascular invasion ***	0.021	3.136	1.190–8.262
**Positive resection margins**	<0.001	7.881	2.575–24.121
Nodal status ****	0.034	3.802	1.104–13.092
**Presence of residual tumor after neoadjuvant treatment**	0.004	5.011	1.662–15.109

HR—hazard ratio (risk ratio); CI—confidence interval; RT—radiotherapy; *—imagistically detected; **—reported versus stage 0 (absence of post-radiotherapy tumor); ***—detected through intraoperative anatomopathological exam; ****—evaluated intraoperatively.

**Table 7:** Independent predictors of survival.

Variable	*p* Value	HR	CI
**Positive resection margins**	0.002	6.646	2.0–22.084
**FIGO stage III post-radiotherapy**	0.003	13.886	2.456–78.506

CI—confidence interval; HR—hazard ratio (risk ratio).

## Data Availability

The patients’ data were obtained from the medical documents of the Bucharest Oncological Institute, and they cannot be publicly available, as they contain personal and confidential data of the patients, but any information about these documents can be obtained on request from the corresponding authors.
